# Multi-Modality Imaging in Dilated Cardiomyopathy: With a Focus on the Role of Cardiac Magnetic Resonance

**DOI:** 10.3389/fcvm.2020.00097

**Published:** 2020-07-02

**Authors:** Panagiota Mitropoulou, Georgios Georgiopoulos, Stefano Figliozzi, Dimitrios Klettas, Flavia Nicoli, Pier Giorgio Masci

**Affiliations:** ^1^Cardiology Department, Queen Alexandra Hospital, Portsmouth, United Kingdom; ^2^School of Biomedical Engineering and Imaging Sciences, King's College London, St Thomas Hospital, London, United Kingdom; ^3^Department of Clinical Therapeutics, National and Kapodistrian University of Athens, Athens, Greece; ^4^First Department of Cardiology, National and Kapodistrian University of Athens, Athens, Greece

**Keywords:** cardiac imaging, dilated cardiomyopathy, cardiac magnetic resonance, non-ischemic cardiomyopathy, heart failure, sudden cardiac death

## Abstract

Heart failure (HF) is recognized as a leading cause of morbidity and mortality worldwide. Dilated cardiomyopathy (DCM) is a common phenotype in patients presenting with HF. Timely diagnosis, appropriate identification of the underlying cause, individualized risk stratification, and prediction of clinical response to treatment have improved the prognosis of DCM over the last few decades. In this article, we reviewed the current evidence on available imaging techniques used for DCM patients. In this direction, we evaluated appropriate scenarios for the implementation of echocardiography, nuclear imaging, and cardiac computed tomography, and we focused on the primordial role that cardiac magnetic resonance (CMR) holds in the diagnosis, prognosis, and tailoring of therapeutic options in this population of special clinical interest. We explored the predictive value of CMR toward left ventricular reverse remodeling and prediction of sudden cardiac death, thus guiding the decisions for device therapy. Principles underpinning the use of state-of-the-art CMR techniques such as parametric mapping and feature-tracking strain analysis are also provided, along with expectations for the anticipated future advances in this field. We also attempted to correlate the evidence with clinical practice, with the intent to address questions on selecting the optimal imaging method for different indications and clinical needs. Overall, we recommend a comprehensive assessment of DCM patients at baseline and at follow-up intervals depending on the clinical status, with the addition of CMR as a second-line modality to other imaging techniques. We also provide an algorithm to guide the detailed imaging approach of the patient with DCM. We expect that future guidelines will upgrade their clinical recommendations for the utilization of CMR in DCM, which is expected to further improve the quality of care and the outcomes. This review provides an up-to-date perspective on the imaging of dilated cardiomyopathy patients and will be of clinical value to training doctors and physicians involved in the area of heart failure.

## Introduction

Heart failure (HF) is one of the leading causes of morbidity and mortality worldwide. In the Western world, ~1–2% of adults develop HF, with the prevalence increasing to ≥10% after the age of 70 (1). Despite therapeutic advances, the individual trajectories of HF course substantially vary, and the clinical outcomes are still disappointing. Early diagnosis, identification of the underlying cause, customized risk stratification, and prediction of response to device or medical therapy are paramount for improving the dismal HF prognosis. To that end, non-invasive imaging techniques play a crucial role by pinpointing preclinical pathophysiological abnormalities, monitoring treatment responses, and attributing personalized risk stratification.

Dilated cardiomyopathy (DCM) is one of the commonest phenotypes in patients diagnosed with HF (2). In this article, we reviewed current evidence on available imaging techniques used in DCM patients. We retrieved published studies which have assessed the clinical value of imaging modalities in the DCM spectrum toward improving diagnosis, optimizing patients' risk stratification, and improving clinical decision-making with salutary effects on healthcare quality and cost burden.

## Definition of Dilated Cardiomyopathy, Prevalence, and Causes

### Definition

**Figure 1:**
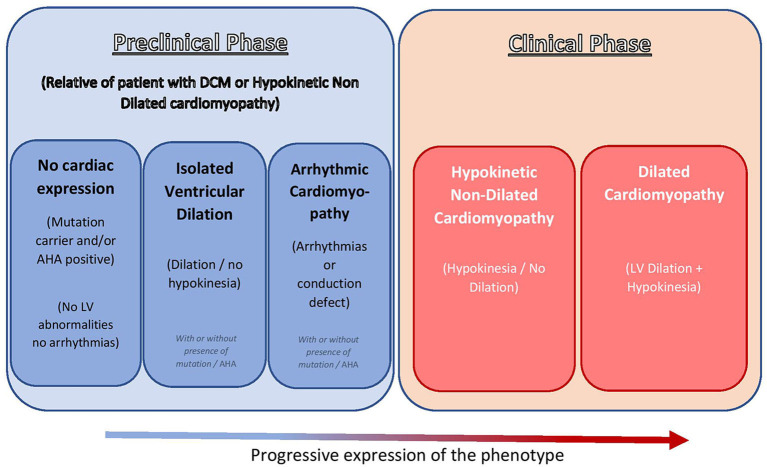
Recent insights in the clinical spectrum of DCM [adapted from Pinto et al. (4) with permission]. DCM, dilated cardiomyopathy; AHA, anti-heart antibody; LV, left ventricle.

The 1995 WHO/ISFC Task Force on the Definition and Classification of Cardiomyopathies initially defined DCM as a spectrum of heterogeneous myocardial disorders characterized by ventricular dilation and depressed myocardial function in the absence of hypertension and valvular, congenital, or ischemic heart disease (3). In 2016, the European Society of Cardiology working group on myocardial and pericardial diseases described DCM as a progressive and often irreversible disorder of the myocardium characterized by left ventricular (LV) or biventricular dilation alongside systolic dysfunction not otherwise explained by abnormal loading conditions such as hypertension and valvular or coronary artery disorders (4). In this revised definition of DCM, the new concept of hypokinetic non-dilated cardiomyopathy (HNDC) was introduced. This new category recognizes that, although systolic dysfunction is typically associated with LV dilatation in DCM, the LV dilatation may occasionally not be seen, as described in Lamin A/C gene mutation carriers (5) and also in some patients without a known genetic cause of DCM (6). Equally, it is recognized that, in several individuals (up to 25% of siblings of patients with familial DCM), a preclinical phase featured by isolated LV dilatation (7, 8) or arrhythmogenic pattern (e.g., early phase of cardio-laminopathy) (9, 10) may occur. HNDC is defined as LV or biventricular global systolic dysfunction [defined as left ventricular ejection fraction (LVEF) <45%] without dilatation, which is not explained by abnormal loading conditions or obstructive coronary artery disease [Figure 1, adapted from Pinto et al. (4)].

### Prevalence

It is difficult to appreciate the exact prevalence of DCM generally and more specifically of genetically mediated DCM (11). An estimate of DCM prevalence as reported by a study performed from 1975 to 1984 in Olmstead County, MN, USA (12), which used echocardiography, angiography, and autopsy to diagnose DCM and examined a European–American population, was 36.5/100,000 people, with a men-to-women ratio of 3:4 (12). The prevalence of the disease varies in studies from different parts of the world, and it has been reported as 8.3/100,000 in a study from England (13), 7.0/100,000 in Italy (14), and 14/100,000 in Japan (15).

Familial DCM represents ~30 to 50% of DCM cases (16–20). A meta-analysis which examined the prevalence of familial DCM and included 23 studies reported an average prevalence of 23% among all DCM cases, ranging from 2 to 65%, depicting the significant heterogeneity due to the diverse diagnostic criteria adopted (21). In patients with familial DCM, approximately 40% have an identifiable genetic cause (19). The pathogenic genetic variants can also be identified in some cases of sporadic DCM, although it is challenging to define the frequency of genetic causes in this population (19).

### Causes

The causes of DCM can be divided into genetic and non-genetic (16). Approximately 40% of the genetic causes have been attributed to rare variants in over 60 genes (1, 22). The most frequently involved genes codify for cytoskeleton or sarcomere proteins. Up to 25 and 18% of cases of familial and sporadic DCM, respectively, have been attributed to truncating variants of titin gene (7). In patients with genetic DCM, cardiac conduction abnormalities may suggest a specific gene defect [e.g., lamin A/C mutations (LMNA) or SCN5A mutations], while elevated serum creatine kinase or muscle weakness points to other genetic substrates (e.g., muscular dystrophy or LMNA mutation) (23). However, a positive family history is the most important clue, and a detailed family history covering at least three generations should be obtained. The current guidance is that genetic testing is recommended only if there is a history of at least two affected family members (24). DCM caused by LMNA mutations has been associated with poor prognosis due to malignant ventricular arrhythmias or rapidly progressive HF (8). In LMNA carriers, non-sustained ventricular tachycardia, male gender, LVEF <45% at presentation, and non-missense mutations are independent predictors of malignant ventricular arrhythmias. As a result, the detection of such mutations may lower the threshold for primary prevention by implantable cardioverter-defibrillator (ICD) implantation (25). In a recently published analysis of 487 patients with familial and non-familial DCM who underwent genetic testing and were followed up for a median of 10.4 years (22), the overall survival was similar in the variant-positive and the variant-negative groups. However, a strong trend toward a higher cumulative incidence of death from HF/heart transplant (HT)/ventricular assist device (*p* = 0.061) and of sudden cardiac death (SCD)/ventricular tachycardia (VT)/ventricular fibrillation (VF) (*p* = 0.062) was observed in variant carriers compared with non-carriers. In the same study, the LMNA carriers demonstrated a higher occurrence of both cardiovascular death/HT (*p* < 0.001) and SCD/VT/VF (*p* = 0.002 vs. variant-negative and *p* = 0.003 vs. remaining carriers). Furthermore, carriers of desmosomal variants also had more frequent arrhythmic events compared with both variant-negative patients (*p* = 0.006) and remaining carriers (*p* = 0.015), while their risk for arrhythmic events was similar to the LMNA subgroup. Interestingly, the correlation of desmosomal variants with SCD/VT/VF was independent of LV dysfunction. Consequently, it was concluded that desmosomal variants are associated with arrhythmia syndromes independently of the left ventricular systolic function, as observed in laminopathies.

The non-genetic causes of DCM include infectious (viral or non-viral) or autoimmune myocarditis, toxic and drug-related causes, nutritional deficiencies, and endocrine and peripartum cardiomyopathy. About 9% of DCM cases are attributed to myocarditis, likely as the consequence of long-lasting inflammatory disease of the myocardium in association with maladaptive post-viral immune-mediated response (9). Peripartum cardiomyopathy develops at the late stage of pregnancy or post-partum, typically within 1 month before the delivery and 5 months post-delivery. Preeclampsia, twin gestation, and advanced maternal age have been recognized as risk factors. A similar distribution of titin truncating variants in women with peripartum cardiomyopathy and DCM patients has been illustrated, raising the suspicion of genetic predisposition (26). As in various acquired causes of DCM, the interaction between genetic predisposition and environmental factors appears to play a crucial role in the development of the disease (6).

Finally, it is worth noting that a significant overlap between the DCM phenotype and other types of cardiomyopathies, such as hypertrophic cardiomyopathy, non-compaction, and arrhythmogenic cardiomyopathy, might be observed. In this direction, a recent study which examined the overlap between non-compaction cardiomyopathy (NCC) with other phenotypes (27) demonstrated that a significant proportion of the affected patients (59%) and their relatives fulfilled the criteria of DCM diagnosis. In the same population, patients with non-compaction and dilated and hypertrophic cardiomyopathy shared a common genetic substrate to a significant degree. For example, gene mutations in MYH7, TTN, and MYBPC3 genes often presented with either NCC, DCM, or an overlapping phenotype. Respectively, arrhythmogenic cardiomyopathy and “arrhythmic forms” of DCM may present ambiguous imaging features, while titin and phospholamban gene mutations (among various mutations) have been found in both clinical entities (28–30). Table 1 summarizes the causes of DCM.

**Table 1:** Causes of dilated cardiomyopathy.

**Idiopathic**		
**Genetic causes**	More than 40 genes have been reported as causal (31)	
**Non-genetic causes**		
	Ischemic heart disease	
	Infiltrative disease	
	Peripartum cardiomyopathy	
	Hypertension	
	Infection	Viral cardiomyopathy
		HIV
		Chagas disease
		Lyme disease
	Connective tissue disease	
	Toxins	Alcohol
		Cocaine
		Medications—particularly chemotherapeutic agents
		Other elements, such as arsenic or cobalt
	Tachycardia-induced cardiomyopathy	
	Stress-induced cardiomyopathy (“Takotsubo”)	
	Nutritional deficiency	Deficiencies in thiamine, selenium, or carnitine
	Endocrine dysfunction	Such as acromegaly, thyroid dysfunction

## The Role of Cardiac Imaging in Dilated Cardiomyopathy

Imaging is crucial for establishing the diagnosis of DCM, as well as for risk stratification, patient management, and treatment monitoring. DCM can have very diverse clinical outcomes, ranging from LV reserve remodeling and recovery of systolic function to acute heart failure, arrhythmias, or SCD. Thus, the therapeutic management of DCM patients necessitates a constant update on the underlying cardiac structural and functional status.

Among the available imaging modalities, transthoracic echocardiography (TTE) is the method of choice for patients with suspected HF, given the broad availability, high portability, and limited cost (23, 32–34). TTE information can be complemented by more advanced modalities, chosen according to their ability to deliver complementary information tailored on specific clinical queries. Cardiovascular magnetic resonance (CMR), nuclear imaging single-photon emission computed tomography (SPECT) and positron emission tomography (PET) and cardiac computed tomography (CCT) are the forefront techniques for implementing DCM diagnosis and patients' workup [Table 2, adapted from Masci and Maestrini (35)]. However, there are practical limitations to the use of each imaging modality (cost, availability, and radiation exposure) which dictate a judicious choice of the optimal imaging technique.

**Table 2:** Different imaging modalities for the evaluation of dilated cardiomyopathy [adapted from Masci and Maestrini (35)].

	**Echo**	**CMR**	**SPECT**	**PET**	**CT**
Chamber dimensions	++	+++	++	++	++
Systolic function	++	+++	++	++	++
Diastolic function	+++	+	+	–	–
Morphologic assessment	++	+++	–	–	–
Dyssynchrony	++	+	+	–	–
Ischemia	++	+++	++	+++	–
Metabolism	–	+	–	+++	–
Tissue characterization	–	+++	–	+	+
Coronary arteries	–	++	–	–	+++
Valve disease	+++	++	–	–	+
Pulmonary hypertension	++	–	–	–	–
Limitations	Acoustic window limitation Operator dependency	AvailabilityMetallic implantsUse of contrast	Radiation exposure Attenuation artifacts	Radiation exposureAvailabilityCost	Radiation exposure Low quality in arrhythmias

*Echo, echocardiography; CMR, cardiac magnetic resonance; CT, computed tomography; PET, positron emission tomography; SPECT, single photon emission computed tomography. The crosses represent how helpful each test is in assessing the index parameter*.

**Figure 2:**
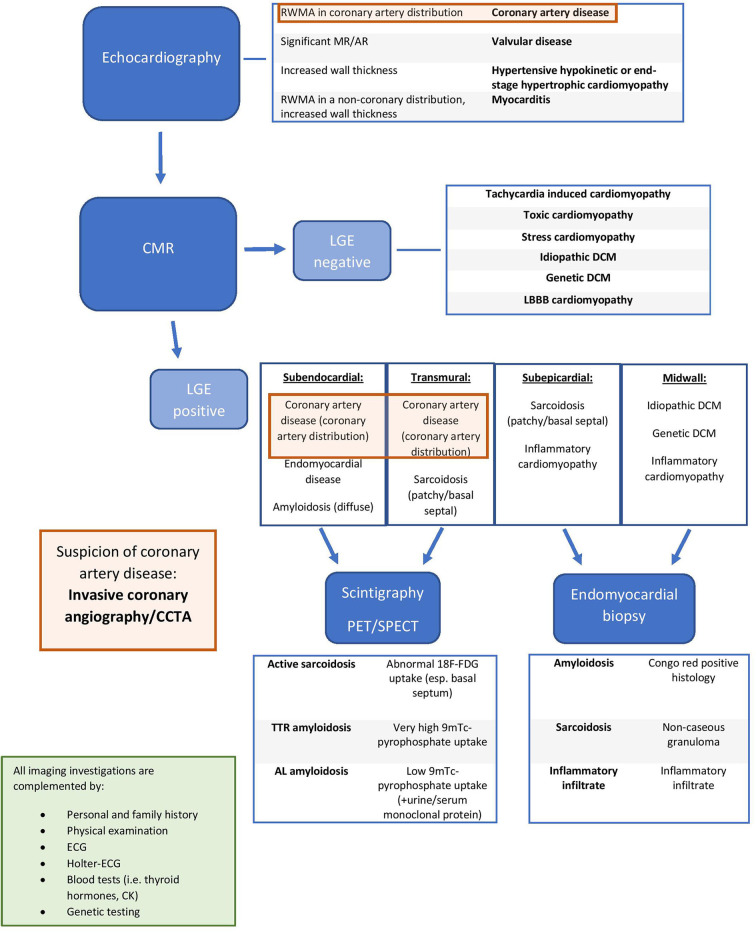
Integrated approach for the differential diagnosis of dilated cardiomyopathy patients [adapted from Porcari et al. (36) with permission]. RWMA, regional wall motion abnormality; MR, mitral regurgitation; AR, aortic regurgitation; LGE, late gadolinium enhancement; DCM, dilated cardiomyopathy; LBBB, left bundle brunch block; CMR: Cardiac Magnentic Resonance; TTR amyloidosis, transthyretin amyloidosis; AL amyloidosis, immunoglobulin light chain amyloidosis; CCTA, Cardiac Computed Tomography Angiography; PET, Positron Emission Tomography; SPECT, Single Photon Emission Computed Tomography; ECG, electrocardiogram, 18F-FDG, 18 fluorodeoxyglucose; 9mTc, technetium-99m.

The major indications for the use of different imaging modalities in DCM are summarized in Table 3. In Figure 2 [adapted from Porcari et al. (36)], we present an approach for the differential diagnosis of patients with DCM [Tables 2, 3; (35)].

**Table 3:** Imaging modalities recommended at the time of diagnosis and during follow-up.

**Imaging modality**	**At diagnosis**	**Follow-up**	
Echocardiography	• Main imaging modality to diagnose left ventricular dilatation and systolic dysfunction • Clues for diagnosis of etiology • Prognostication (left/right ventricular function; degree of mitral regurgitation, presence of diastolic impairment)	• Prognostication (left/right ventricular systolic function improvement, mitral regurgitation improvement: left ventricular restrictive filling pattern improvement)	Main imaging technique during follow up—should be repeated at regular intervals
CMR	• Accurate assessment of volumes and systolic function • Differential diagnosis • Identification of cause • Prognostic stratification, including risk of sudden cardiac death (right ventricular involvement, late gadolinium enhancement)	• Increasingly used for prognostication	Role of CMR during follow-up needs to be further assessed
CT coronary angiogram	• Identification of cause (exclusion of ischemic heart disease—to be used in patients with low pre-test probability for coronary artery disease)	Not used	
PET/SPECT	• Tissue characterization—can aid in the diagnosis of the cause of left ventricular dysfunction (for example, sarcoidosis or cardiac amyloidosis), which has implications on treatment and prognostication	In the case of sarcoidosis, 18F-FDG PET is used to monitor the response to steroids	

*CMR, cardiac magnetic resonance; CT, computed tomography; PET, positron emission tomography; SPECT, single photon emission computed tomography; 18F-FDG, 18F-fluorodeoxyglucose*.

### Echocardiography

Two-dimensional (2D) transthoracic echocardiography (TTE) is the first-line imaging method as it provides information on chamber dimensions and morphology, systolic and diastolic function, as well as presence and severity of valve disease, in a broadly available, non-invasive, and cost-effective manner (37). The diagnostic criteria for DCM have relied on the identification of a LVEF <45% and/or a fractional shortening <25%, in association with a LV end-diastolic dimension >112% predicted value corrected for age and body surface area (37).

TTE also provides a comprehensive assessment of cardiac anatomy and hemodynamics. A major advantage of TTE is its unique ability for non-invasive hemodynamic assessment, which renders this tool the modality of choice for studying valvular heart disease (e.g., functional mitral regurgitation associated with DCM phenotype) and for gauging the ventricular diastolic function.

Aside from its crucial role in diagnosis, TTE is used to identify high-risk features and to assess prognosis. LV systolic dysfunction has long been regarded as the main determinant of prognosis in DCM patients (38). An accurate quantification of LV function is regarded as the main feature guiding patient management and subsequent treatment, including the indication for ICD, resynchronization therapy, or discontinuation of cardiotoxic chemotherapy. The apical biplane method of discs (modified Simpson's rule) is the recommended technique for measuring LV volumes and EF, and contrast agents may be administered to better delineate the endocardial border when image quality is sub-optimal (39). Three-dimensional (3D) TTE may overcome the limitations inherent to 2D TTE with respect to LV volumes and EF estimates, and it should be performed when available in experienced laboratories. Indeed the reproducibility of LV volume calculation and EF has been shown to improve with 3D echocardiography (40). Poor acoustic window and inter-operator variability remain as limiting factors.

Right ventricular (RV) dysfunction is associated with worse functional status and outcome in DCM; thus, the assessment of the RV systolic function should be part of any standard echocardiographic investigation. However, the quantification of RV function is challenging due to its complex 3D shape. The 2D TTE criteria for RV systolic dysfunction are: RV fractional area change (FAC) of <35%, tricuspid annular plane systolic excursion (TAPSE) of <17 mm, and tricuspid annulus S velocity <9.5 cm/s (derived from tissue Doppler imaging) (41). A TAPSE <14 mm has been found to correlate with an adverse prognosis in patients with DCM (42). RV systolic dysfunction assessed by RV fractional area change (defined as RV fractional area change of <35%) has been associated with increased risk of death or cardiac transplantation (43). Further prognostic information can be provided by estimating the pulmonary artery pressure, which is calculated by measuring the tricuspid regurgitation velocity and assessing the inferior vena cava size and inspiratory collapse (44).

With respect to concomitant valvular disease, DCM patients may develop secondary mitral regurgitation (MR) as a result of the apical tethering of the leaflets, annular dilatation, and/or ventricular dyssynchrony. TTE is considered the imaging modality of choice again for gauging MR severity and progression (45).

Furthermore, TTE can be employed for estimating the presence and the extent of mechanical dyssynchrony in the failing heart, and therefore it can serve as an aid to patient selection for cardiac resynchronization therapy (CRT), in addition to clinical and electrocardiographic parameters. Under the same prism, serial TTEs can provide useful feedback for CRT optimization in non-responders. Several techniques have been described, including M-mode (46, 47), Doppler echocardiography (48, 49), and tissue Doppler imaging (50–53).

Stress echocardiography (SE) can provide useful information by assessing the presence of contractile reserve (which is defined as improvement in wall motion score, fractional shortening, or EF during stress) (54). It is more commonly assessed during dobutamine infusion (10–40 mcg/kg/min); however, exercise SE can also be used. Exercise SE protocols can be used in DCM patients to assess systolic and diastolic reserve, pulmonary pressures, and dynamic MR (55). The presence of contractile reserve irrespective of stressor (dobutamine/exercise) is associated with better prognosis (80% lower mortality and lower rates of cardiovascular events and hospitalization) (56). It may also help in screening for pre-clinical DCM (e.g., asymptomatic LV dysfunction), such as in patients who have received anthracycline chemotherapy (57). SE has been used to guide therapeutic decisions in candidates to cardiac transplantation (58). Additionally, dobutamine SE has been validated to identify inducible myocardial ischemia and viability (59).

A promising TTE technique is speckle tracking echocardiography (STE), which enables a thorough assessment of cardiac mechanics and deformation circumventing some of the limitations inherent to LVEF. Indeed global longitudinal strain (GLS) has emerged as one of the most useful parameters for improving risk stratification in DCM patients, and it has been shown to be superior to other echocardiographic parameters in predicting all-cause mortality in patients with HF of various causes (60). It has also been used to assess LV dyssynchrony (mechanical dispersion), and it has been shown to constitute a good marker of arrhythmias in the non-ischemic cardiomyopathy population (61). The prognostic value of GLS has been demonstrated both in chronic (62) and in acute (63) HF.

GLS is an emerging tool in the early detection of subclinical LV systolic dysfunction, especially before the LVEF is affected, given its higher sensitivity in the systolic function assessment (64). This has important implications particularly in two patient groups. Firstly, GLS is very promising for the early detection of cancer therapy-related cardiac dysfunction. A recent meta-analysis, which included 21 studies and 1,782 patients treated with anthracyclines with or without trastuzumab, demonstrated that GLS has a good prognostic performance for the detection of cancer therapy-related cardiac dysfunction (65). Secondly, GLS appears to have better diagnostic and prognostic performance than LVEF in the assessment of LV systolic dysfunction in relatives of DCM patients during familial screening. In a recent study (66), abnormal GLS predicted deterioration in LVEF and carried a worse prognosis (in terms of cardiac hospitalizations and death) in relatives of DCM patients [hazard ratio (HR) 3.37, 95% confidence interval (CI) 1.11–10.2]. This finding may have significant implications on the screening of relatives of DCM patients.

Despite the promising evidence that has been discussed, a rather high inter- and intra-observer variability challenges the applicability of STE. Furthermore, GLS depends on image quality, and therefore it cannot be used in a significant proportion of patients who have suboptimal echocardiographic acoustic windows.

### Nuclear Imaging

Nuclear imaging techniques can be used in DCM patients to detect myocardial perfusion defects, myocardial viability, and inducible ischemia. However, the role of these techniques for the diagnosis of DCM *per se* is still limited (67). SPECT is useful for excluding myocardial ischemia and providing prognostic information, particularly when gated SPECT is utilized for the assessment of LV volumes and function. PET alone or in combination with cardiac CT is a valid but expensive alternative for detecting myocardial ischemia. There is evidence that assessing myocardial blood flow and myocardial blood flow reserve using PET has a prognostic value in both ischemic and non-ischemic cardiomyopathy (68).

In patients with a new diagnosis of DCM, nuclear imaging complements other imaging modalities for an insightful search of rather uncommon etiologies including sarcoidosis or amyloidosis (69). The typical sarcoidotic lesion can be detected by technetium 99m, thallium 201, or gallium-67 radionuclide SPECT (70). Currently, 18F-fluorodeoxyglucose (18F-FDG) PET is recognized as the most sensitive diagnostic tool for identifying the inflammatory areas of sarcoidosis, alongside the detection of extra-cardiac lesions, thus enhancing the diagnosis of this condition (71). Nonetheless, it should be acknowledged that the suboptimal glucose metabolism suppression of the myocardium hampers the interpretation of 18F-FDG images. Inflammatory specific tracers such as somatostatin receptor–ligands (72, 73) and quantitative radiotracer uptake (74) are likely to implement the diagnostic accuracy of PET in the diagnosis of inflammatory myocardial disease presenting with a DCM-like phenotype.

In cardiac amyloidosis [particularly amyloid transthyretin (ATTR) amyloidosis], 99mTc-pyrophosphate (99mTc-PYP), 99mTc-methylene diphosphonate (99mTc-MDP), and 99m Tc-3,3-diphosphono-1,2-propanodicarboxylic acid (99mTc-DPD) SPECT have been proposed as accurate diagnostic techniques to single out amyloidotic cardiomyopathy (75, 76). Their role is crucial in the context of the recent development of novel treatments for ATTR amyloidosis. Furthermore, amyloid PET imaging has been employed with very promising results for amyloid cardiomyopathy irrespective of the type of amyloid (AL or ATTR) (77, 78).

^123^I-metaiodobenzylguanidine (^123^I-MIBG) SPECT is another non-invasive technique which adds to the DCM risk stratification and prognosis (79, 80). ^123^I-MIBG is a norepinephrine analog and can be used to tailor treatment and improve the risk stratification of heart failure patients by assessing sympathetic innervation. To that end, Yamazaki et al. (81) showed that (^123^I-MIBG) SPECT is associated with the severity of DCM, and it can be used to predict the applicability of beta-blockade therapy, guide the dose of beta-blocker, and convey prognostic information. Several studies have also implemented (^123^I-MIBG) SPECT to assess regional denervation and its value in predicting arrhythmic events. Overall, the extent of ^123^I-MIBG SPECT appears to be proportional to the risk of ventricular tachyarrhythmia (82–86). The prospective ADMIRE-HF study (87) included a total of 961 patients with symptomatic heart failure and LVEF ≤35% (both ischemic and non-ischemic etiology) and quantified the sympathetic activity on ^123^I-MIBG SPECT as the heart/mediastinum uptake ratio [H/M] on 4-h delayed planar images. A H/M ratio of ≥1.60 was predictive of HF progression (HR 0.49, 95% CI 0.32–0.77), potentially life-threatening arrhythmic events (HR 0.37, 95% CI 0.16–0.85), and cardiac death (HR 0.14, 95% CI 0.03–0.58). The ADMIRE-HFX study (88) extended the period of follow-up of the same patients and found a significant benefit in reclassifying HF patients with the addition of the H/M ratio to a prognostic model including BNP and LVEF.

### Invasive Coronary Angiogram and Cardiac Computed Tomography

Invasive coronary angiography is recommended in patients with LV systolic dysfunction and typical angina or evidence of myocardial ischemia (39). CCT is a valuable alternative for coronary anatomy assessment, particularly in subjects with low–intermediate likelihood of coronary artery disease (CAD) (89) or in patients with a high suspicion of constrictive pericarditis as a potential cause of HF (69). Felker et al. (90) classified patients with LV systolic dysfunction as ischemic or non-ischemic based on the extent of obstructive CAD underpinned by coronary angiograms. Ischemic etiology has been shown to carry a worse prognosis in patients with LV dysfunction in a variety of studies (38, 91, 92), while the etiology of HF also determines the decision to pursue revascularization and may affect the response to therapy (93). Therefore, the accurate distinction between ischemic and non-ischemic cardiomyopathy is paramount.

In this context, CCT provides a useful means for ruling out CAD in patients with LV systolic dysfunction, given its high negative predictive value (94). With regards to left main and/or three-vessel CAD in symptomatic patients, CCT has been shown to accurately exclude this with a negative predictive value of 99% (CI 98–99%). CCT has been validated for detecting CAD in patients with dilated cardiomyopathy (89). Bhatti et al. (95) demonstrated that it can be used to exclude an ischemic etiology in patients with cardiomyopathy of undetermined cause (sensitivity 98% and specificity 97%). As such, it is a very useful tool for reducing the rate of negative invasive coronary angiography in this patient population.

The European (34) and the American (24) guidelines indicate that CCT should be considered in patients with HF and low to intermediate pre-test probability of CAD or those with equivocal non-invasive stress tests in order to rule out coronary artery stenosis (class IIb, level of evidence C), while invasive coronary angiography should be reserved for patients with HF, intermediate to high pre-test probability of CAD, and presence of ischemia on non-invasive stress tests, who are thus considered suitable for potential coronary revascularization (class IIa, level of evidence C).

Furthermore, CCT using retrospective triggering can provide anatomical and functional information, such as LV and RV volumes and EF, as well as assessment of regional wall motion abnormalities. LV volumes, mass, and EF measurements by CCT have been shown to correlate strongly with echocardiography (96, 97) and CMR (97, 98). Moreover, regional wall motion can be evaluated with good accuracy and precision (96, 99, 100), paralleling those of CMR (101). Given the specific attenuation of the diverse tissues and its high spatial resolution, CCT enables to pinpoint small areas of myocardial fat infiltration, orienting the diagnosis of the etiology of DCM as prominent trabeculations and areas of non-compaction suggest non-compaction cardiomyopathy, while fat in the RV wall and abnormal RV wall motion may suggest arrhythmogenic RV dysplasia (102).

Currently, two techniques for the assessment of myocardial ischemia using CCT are emerging into clinical practice: stress perfusion CT and CT-derived fractional flow reserve (CT-FFR).

Stress perfusion CT (CTP) can be performed immediately after the traditional CCT angiography, using conventional pharmacologic stress agents. CTP images can be either static or dynamic. One of the main advantages of dynamic CTP over static is the quantification of the myocardial blood flow. Dynamic CTP uses serial image acquisition to monitor the transition of contrast in the arterial blood pool and the myocardium. CTP has shown better diagnostic performance than SPECT for the diagnosis of significant disease on invasive angiography, driven in part by the higher sensitivity for left main and multivessel disease (103). A recent meta-analysis on static CTP including almost 1,200 patients showed that CTP improves specificity compared with CCT angiography alone (104). Additionally, dynamic CTP imaging allows the quantification of absolute cardiac functional reserve.

With regards to CT-FFR, several trials have shown that its addition improves diagnostic accuracy compared with CCT angiography alone (105–108). Furthermore, data from the PLATFORM study suggest that a CT-FFR-guided strategy is associated with equal diagnostic performance but is more cost-efficient at 1 year compared with invasive angiography (109).

## Cardiac Magnetic Resonance

CMR has emerged as an indispensable diagnostic tool in the workup of DCM patients, given its ability to provide accurate and reproducible measurements on biventricular volumes, mass, and function, as well as detailed morphology information, overcoming most of the limitations inherent to other imaging modalities. Accordingly, CMR is the best imaging modality in patients with non-diagnostic or doubtful 2D-echo (class I, evidence C) (39). CMR is regarded as the gold standard with respect to accuracy and precision of ventricular volumes, mass, and wall motion.

Furthermore, CMR has the unique ability to non-invasively characterize the composition of the myocardium, making it an excellent diagnostic tool to differentiate the etiologies of DCM. The lack of radiation exposure and the safety of non-linear gadolinium-based contrast agents render CMR suitable and safe for serial scans in adults and in pediatric subjects.

The limitations of CMR include lack of availability, inability to image patients with specific contraindications, and cost (39, 110). There are also concerns regarding the risk of nephrogenic systemic fibrosis as a result of the use of gadolinium in patients with severely impaired renal function. Nonetheless, a recent meta-analysis (111) concluded that the risk of nephrogenic systemic fibrosis from the use of cyclic gadolinium-based contrast agents in patients with chronic kidney disease stages 4 and 5 is likely less than 0.07%. Therefore, contrast-enhanced CMR scans are likely to outweigh the risk of nephrogenic systemic fibrosis in these patients (Table 4).

**Table 4:** Cardiac magnetic resonance report of patients evaluated for dilated cardiomyopathy.

**Parameters**	**Left ventricle**	**Right ventricle**	**Left atrium**	**Right atrium**	**Others**
Volumes/BSA	+	+			
Stroke volume/BSA	+	+			
Mass/BSA	+	(+)			
Regional systolic function	+	+			
Global systolic function	+	+			
End-systolic surface/BSA (four-chamber view)			(+)	(+)	
Morphology(maximal ED wall thickness)	(+)				
Cardiac morphology and coronary angiography					(+)
Native T1 mapping/ECV	+				
T2 mapping	+				
T2^*^	(+)				
Thrombus	+	+			
LGE presence and location	+	+	+		

*BSA, body surface area; ED, end-diastolic; LGE, late gadolinium enhancement; LGE pattern, non-ischemic (a: patchy, b: mid-wall, c: sub-epicardial) and ischemic [a: sub-endocardial (transmurality <50%), b: transmural (transmurality ≥50%)]; +, recommended; (+), optional*.

* *CMR sequence*.

### Diagnostic Performance

**Figure 3:**
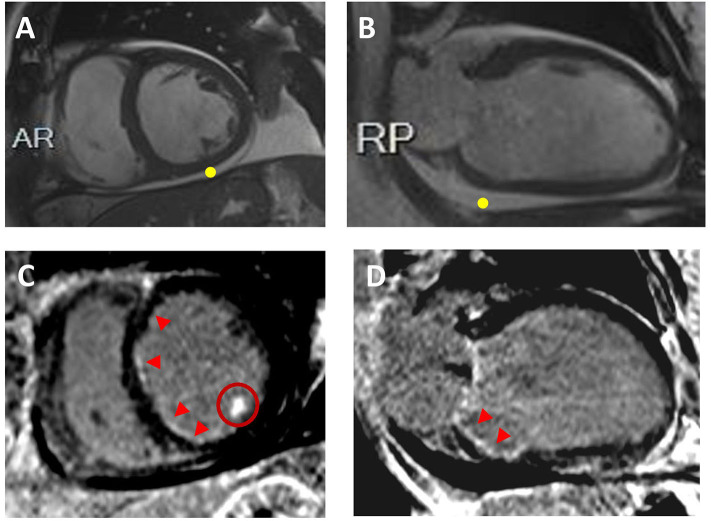
Ischemic Cardiomyopathy. Sixty-two year-old male with multiple cardiovascular risk factors including diabetes, presented with decompensated heart failure. CMR revealed biventricular dilatation and systolic disfunction. Representative short-axis **(A)** and 2-chamber **(B)** cine images are displayed without evidence of LV wall thinning; pericardia! effusion is visualized (yellow point). On the corresponding short-axis **(C)** and 2-chamber **(D)** post-contrast images, sub-endocardial late gadolinium enhancement is visualized in the right and left coronary artery system (red arrowheads). Hyperenhancement of the inferolateral papillary muscle is also seen (**C**; red circle). Coronary angiograms showed obstructive coronary artery disease.

In the workup of patients with LV systolic dysfunction, it is crucial to make the distinction between ischemic and non-ischemic etiologies as the treatment strategy and the prognosis of these two entities diverge (34). CMR is highly effective in detecting the causes of LV dysfunction in newly diagnosed HF patients with unclear etiology (Figure 3).

The late gadolinium enhancement (LGE) technique is currently the non-invasive gold standard for the identification and the quantification of myocardial scar. LGE is used to evaluate replacement fibrosis (i.e., myocardial scar), providing key information with respect to etiology (e.g., post-myocarditis, DCM, or ischemic cardiomyopathy) and clinical outcome (112). Patients with an ischemic etiology show subendocardial/transmural LGE within one or multiple coronary artery vascular territories (*ischemic pattern*), whereas those with a non-ischemic cause of LV dysfunction have either no LGE or LGE with *non-ischemic pattern* (i.e., mid-wall/sub-epicardial or patchy distribution). However, up to 13% of patients with LV dysfunction and no significant coronary artery disease at invasive coronary angiography have areas of LGE with an ischemic pattern (113, 114). In these patients, the extent of ischemic LGE is often small, and it cannot explain the severity of LV dilatation and dysfunction (*DCM with bystander infarct*), although in a small but not negligible number of cases the ischemic LGE extent may be large enough to explain the degree of LV dysfunction (*ischemic cardiomyopathy with unobstructed coronary arteries)* (113, 114). The presence and the extent of LGE have important implications on patients' risk stratification (discussed below).

Furthermore, CMR can incorporate whole-heart coronary angiography. Current evidence indicates that CMR coronary angiography has a good negative predictive value in excluding proximal obstructive coronary artery disease, paralleling the CCT angiography performance (115). However, it should be acknowledged that this technique is currently used in research centers and is not part of the standard CMR protocol utilized in patients with LV systolic dysfunction.

The information acquired by LGE can be complemented by first-pass perfusion CMR for assessing the ischemic burden. The latter, alongside information of myocardial viability, can differentiate ischemic from non-ischemic cardiomyopathy and guide patients' management, supporting the physician in the choice of the best treatment (coronary revascularization vs. optimal medical therapy). First-pass perfusion CMR has been shown to have high diagnostic accuracy, with an area-under-the-curve of 0.95 (0.91–0.99), similar to the diagnostic accuracy of PET imaging (diagnostic accuracy, 0.93) and outperforming SPECT (diagnostic accuracy, 0.82) (116). Multiple studies have assessed the diagnostic accuracy of CMR perfusion imaging, and recent meta-analyses have provided an extensive overview (116–119).

Mapping techniques enable to implement myocardial tissue characterization beyond LGE. Mapping allows to derive T1, T2, and T2^*^ values of the myocardium, which represent the intrinsic properties of the tissue and are modified by the disease. There are recommendations for the standard imaging protocol for myocardial tissue characterization (120); however, this can be modified according to the clinical suspicion and the findings (121).

**Figure 4:**
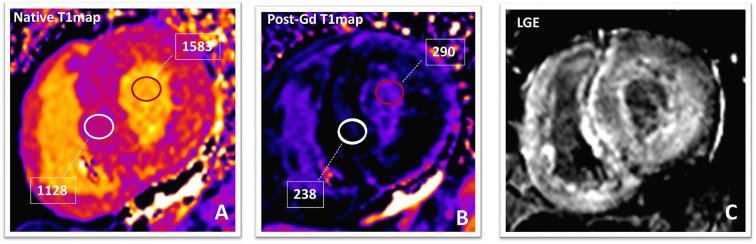
Amyloidosis. Native **(A)** and post-contrast **(B)** T1-maps and phase-sensitive inversion-recovery **(C)** images in a patients with TTR amyloidosis. The native T1value (1,129 ms; normal value in our laboratory is 899–1,027 ms) and ECV (70%; normal value <30%) of the myocardium are markedly elevated suggesting a high amyloid burden. After 10 min from Gadolinium-based contrast-agent bolus, the T1value of the myocardium (238 ms) is lower than that of blood pool (290 ms). Post-contrast phase-sensitive inversion-recovery image **(C)** shows diffuse LGE with relative sparing of the mid-wall of the interventricular septum (“zebra-like” pattern). LGE becomes transmural in the LV inferolateral segment, confirming high amyloidotic burden. TTR, transthyretin; ECV, extracellular volume fraction; LGE, late gadolinium enhancement; LV, left ventricle.

Pre- and post-contrast T1 mapping, coupled with actual or synthetic (derived from pre-contrast T1 value of the LV blood pool) hematocrit, enables one to gauge the extracellular volume (ECV) of the myocardium. Native (pre-contrast) T1 mapping and ECV have been employed to quantify cardiac amyloid burden both in ATT and in AL amyloidosis (122). In the absence of causes known to expand the cardiac interstitum (edema or amyloid deposits), ECV is a valuable biomarker of interstitial fibrosis (123). Specifying myocardial compartmental involvement may then implicate cellular/molecular disease pathways for treatment and targeted pharmaceutical development and, above all, highlight the role of the cardiac-specific pathology in heart failure among myriad other changes in the heart and beyond. For instance, it has been suggested that interstitial fibrosis is involved in the genesis of re-entry circuits and in the generation of focal tachycardias (124). Therefore, an assessment of the interstitial fibrosis offers potential for improving the risk stratification of DCM patients (125). Native (pre-contrast) T1 times and ECV fraction correlate with the degree of interstitial fibrosis in DCM [(123, 126); Figure 4].

Furthermore, T2 mapping can detect myocardial edema and consequently active inflammation as T2 relaxation is directly proportional to tissue water content. A recent meta-analysis reported pooled weighted sensitivity, specificity, and diagnostic accuracy of T2 mapping of 63, 76, and 68%, respectively, for the detection of acute myocarditis (121).

T2^*^ (star) relaxation mapping is the method of choice for the non-invasive assessment and quantification of cardiac iron. Iron deposition in the myocardium can be due to a variety of hematological diseases (such as thalassemia, hemolytic anemia, and sideroblastic anemia) or other conditions. Iron overload impairs the left ventricular systolic function, occasionally leading to DCM-like phenotype. Consequently, T2^*^ values can be useful when considering the differential diagnosis of DCM. A T2^*^ value of the myocardium equal or below 10 ms is associated with severe iron overload, and 98% of thalassemic patients with a T2^*^ in this range developed overt HF at 1-year follow-up (127). Accordingly, it is current practice to refer these patients to rapid hematological workup for iron chelator therapy to be started and then monitor the treatment response by repeating CMR at short intervals. Furthermore, native (pre-contrast) T1 mapping holds the potential for improved detection of mild iron loading (128).

**Figure 5:**
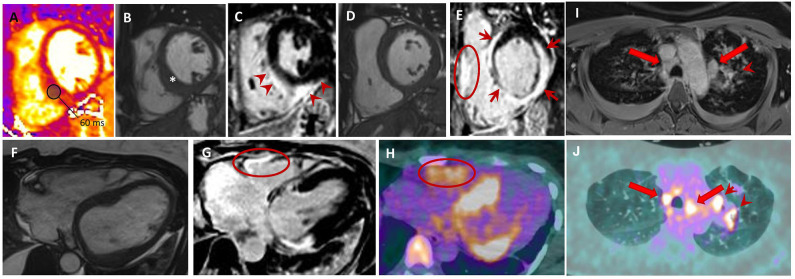
Sarcoidosis. Forty-six year old woman with aborted SCD and evidence LVEF = 46% on TTE. Short-axis T2-mapping **(A)**, cine **(B,D)** and post-contrast phase-sensitive inversion recovery **(C, E)** images show a focally thickened interventricular septum (**B**; *) with an increased T2 value (**A**; T2 value of the inferior septum is 60 ms; normal value <54 ms). Post-contrast **(C,E)** images display LGE **(E)** of the LV interventricular septum, inferior and lateral walls with sub-epicardial to transmural patterns (**E**; red arrows); RV free wall LGE is also seen (**E**; red circle). Axial cine **(F)**, phase-sensitive inversion-recovery **(G)**, and 18F-FDG PET **(H)** images showing an excellent match between LGE positive regions, including the RV free-wall, and those with FDG uptakes. Upper thorax axial non-ECG-triggered post-contrast Tl-weighted **(I)** and 18F-FDG PET **(J)** showing mediastinal lymph nodes (red arrows) and left pulmonary lesions (arrowheads). SCD, sudden cardiac death; LVEF, left ventricular ejection fraction, TTE, transthoracic echocardiogram; LGE, late gadolinium enhancement; RV, right ventricular; 18F-FDG PET, 18 fluorodeoxyglucose positron emission tomography; ECG, electrocardiogram.

Sarcoidosis is an important differential in the etiological workup of DCM patients as this diagnosis changes the management and prognosis radically. A recent meta-analysis, which included eight studies and 649 patients, reported that CMR can diagnose cardiac sarcoidosis with a sensitivity of 0.93% (95% CI, 0.87–0.97) and specificity of 0.85 (95% CI, 0.68–0.94). The most common CMR findings of cardiac sarcoidosis are a variable pattern of delayed gadolinium enhancement with typically mid-wall and or epicardial enhancement, mainly involving the basal segments of the myocardium and in particular the septum and the lateral wall, nodular mid-wall hyper-intense foci on edema-sensitive sequences, as well as areas of focal thickening of the myocardium (Figure 5).

### Overall Prognostication of Adverse Events in DCM

Besides providing insights into the differential diagnosis of DCM, CMR can facilitate the prediction of the trajectory of the disease.

In this context, mid-wall fibrosis as detected by LGE conveys robust prognostication in DCM. Large cohort studies and meta-analysis have clearly pointed out that the occurrence of myocardial scar as detected by LGE is an independent prognosticator for “hard” events including all-cause mortality, future hospitalization, and sudden cardiac death, with additional prognostic value to traditional risk features including LVEF (129–132). A meta-analysis including 1,488 patients with a mean follow-up of 30 months demonstrated that patients with LGE had increased overall mortality, heart failure hospitalizations, and SCD/aborted SCD compared to those without LGE (133). In parallel, a larger meta-analysis (134) which included 34 studies and 4,554 patients showed that LGE presence was associated with cardiovascular mortality [odds ratio (OR, 3.40; 95% CI, 2.04 to 5.67)], ventricular arrhythmic events (OR, 4.52; 95% CI, 3.41 to 5.99), and rehospitalization for HF (OR, 2.66; 95% CI, 1.67 to 4.24). Notably, mid-wall fibrosis retains its prognostic value when considered as a continuous variable, supporting the concept that the extent (and not only the presence) of fibrosis consists a prognostic marker (132).

Recent studies have investigated the value of T1 mapping in risk stratification. Chen et al. assessed 130 patients referred for primary-prevention ICD implantation, among whom 59 had non-ischemic cardiomyopathy. He found that the native T1 values were significantly higher in patients who achieved the primary endpoint of appropriate ICD therapy or sustained ventricular arrhythmia (135). Puntmann et al. (136) assessed 637 patients with non-ischemic DCM and found that T1 mapping indices (native T1 and ECV), as well as the extent of LGE, were predictive of all-cause mortality and the composite endpoint of HF mortality and hospitalization.

Notably, ECV has been shown to hold prognostic value incremental to LGE or native T1 mapping. Barison et al. (137) were the first to demonstrate that myocardial ECV may predict outcomes (composite of cardiovascular death, HF hospitalizations, and appropriate defibrillator intervention) in DCM. Vita et al. (138) studied a non-ischemic HF population and demonstrated that mean ECV was strongly associated with major adverse cardiac events. Abnormal ECV measurements yielded a 2.8-fold increased odd of adverse outcome, independently of age, sex, functional class, and LVEF.

Feature tracking (FT) strain analysis obtained from cine imaging represents another promising tool for improving DCM patients' risk stratification. In parallel with STE, FT can be obtained by post-processing algorithm on routinely acquired cine imaging without the need for dedicated pulse sequences. There is evidence to suggest that FT parameters can predict survival in DCM and refine risk stratification beyond clinical parameters, biomarkers, LVEF, and LGE. One of the first studies to assess FT in the DCM population (139) demonstrated that global and mean longitudinal strain conveyed independent prognostic value surpassing that of NT-proBNP, LVEF, and LGE. Indeed it was demonstrated that preserved GLS carried excellent prognosis even in patients with LVEF <35% and in those with LGE, while mean longitudinal strain was a more valuable prognostic marker than functional class, LVEF, or LGE (HR = 5.4, *P* < 0.01). In a more recent large study (140) including 1,012 ischemic and non-ischemic cardiomyopathy patients, GLS was incremental in risk stratification with respect to LVEF and LGE extent. In patients with DCM, after adjusting for clinical and imaging risk factors such as LVEF and LGE, GLS was found to be significantly associated with all-cause of death (HR, 2.101 per percent; *p* < 0.001).

Finally, the assessment of RV systolic function in DCM has been shown to improve risk stratification for all-cause mortality or cardiac transplantation (CT). In one study, Gulati et al. (2) prospectively studied 250 consecutive DCM patients. The presence of RV systolic dysfunction, which was defined by RV EF ≤45% measured by CMR, resulted in a higher risk of all-cause mortality or CT during a median follow-up period of 6.8 years (HR, 5.90; 95% CI, 3.35–10.37). On multivariable analysis, RV systolic dysfunction remained a significant independent predictor of the primary endpoint of all-cause mortality or CT (HR, 3.90; 95% CI, 2.16–7.04), as well as secondary outcomes of cardiovascular mortality or CT, and HF hospitalization, cardiac death, or CT.

### Prediction of Left Ventricular Reverse Remodeling

Prior studies have shown that nearly 40% of newly diagnosed DCM patients experience LV reverse remodeling (LVRR) at mid-term follow-up. The latter consists of a substantial decrease of LV end-diastolic volume alongside an increase of LVEF, and this favorable phenomenon is a strong and independent predictor of long-term outcome (141, 142).

In this context, CMR holds promises in showing that DCM patients without mid-wall LGE are more likely to experience LVRR, as compared to those with LGE, irrespective of the severity of clinical status, LV dilatation, and dysfunction at initial evaluation (142). A large meta-analysis (134) including 4,554 patients showed that the absence of LGE was a very strong predictor of LVRR (OR, 0.15; 95% CI, 0.06 to 0.36) (143).

Additional techniques such as native T1 mapping and ECV are promising biomarkers which might help to refine the prognostic risk stratification and the prediction of LVRR (144, 145).

However, despite the existing evidence for the above techniques, each one of them in isolation is probably too weak to accurately predict LV reverse remodeling. Therefore, an integrated approach considering the imaging parameters as complementary to each other and using them in combination with clinical observations can lead to the elaboration of multi-parametric scores for a more accurate prediction of LVRR and long-term outcomes (146). There is increasing evidence that an early CMR should be incorporated in the initial workup of non-ischemic cardiomyopathy patients, both for its diagnostic properties and for its early prognostic stratification. CMR can be equally repeated during the follow-up of DCM patients to provide information on any changes on the above parameters and to update the assessment of LV volumes and EF.

### Prediction of Risk of Sudden Cardiac Death

Primary prevention ICD is currently recommended for symptomatic (NYHA classes II and III) patients with LVEF ≤35% despite ≥3 months of optimal medical therapy (class I, level of evidence B) (34). This criterion has been abundantly questioned considering the low sensitivity and specificity for identifying high-risk patients, the non-negligible inappropriate ICD interventions, the ICD placement-related complications, and the significant cost burden. It is also questionable whether this time window is adequate for safe and cost-effective decision-making. Thus, it is paramount to identify which patients are at significant risk of SCD in order to individualize primary-prevention ICD insertion. This is particularly true after the results of the DANISH trial, which showed no benefit of ICD insertion in non-ischemic cardiomyopathy patients fulfilling the current criteria for primary prevention of SCD. While ICD was effective in reducing SCD, this salutary effect did not translate into a significantly lower rate of death from any cause than usual clinical care (147).

As already mentioned, several studies have demonstrated that mid-wall fibrosis on LGE can contribute to the prognostication of sudden cardiac death in DCM (129, 132, 133, 143, 148). A large study (132) followed up 472 patients with DCM of all severities for a median of 5.3 years. After adjustment for other prognostic factors, the presence and the extent of mid-wall fibrosis predicted the arrhythmic composite endpoint, as well as all-cause mortality. The addition of mid-wall fibrosis to LVEF significantly led to risk reclassification for SCD/aborted SCD, with 29% of patients being correctly reclassified after the addition of mid-wall fibrosis to a model including LVEF alone. These findings have been confirmed in three meta-analyses including sizeable samples of DCM patients (133, 148–150).

Two recently published studies have attempted to shed further light on the hypothesis that the presence of a LV scar (as detected by LGE on CMR) should guide patient selection for implantation of primary-prevention ICD, with controversial results. In the first one, a prospective non-randomized study, Gutman et al. (151) evaluated 452 non-ischemic cardiomyopathy patients with LVEF <35% on optimal medical therapy who met the criteria for ICD insertion. After a median follow-up period of 37.9 months, ICD implantation in patients without LV scar on CMR did not appear to improve all-cause mortality (HR, 1.22; 95% CI, 0.53–2.78) or cardiovascular death (HR, 1.64; 95% CI, 0.46–5.89). In contrast, in patients with LGE, ICD was beneficial in reducing all-cause mortality (HR, 0.45; 95% CI, 0.26–0.77) and cardiovascular death (HR, 0.51; 95% CI, 0.27–0.97). The second study, the DANISH-MRI study, was a pre-specified sub-study of the above-mentioned DANISH trial (152). Under a prospective randomized design, 252 patients with non-ischemic cardiomyopathy and indication for primary-prevention ICD on optimal medical management were assessed with CMR. The authors concluded that ICD implantation did not impact all-cause mortality, irrespective of the presence of LV scar (HR for patients with LV scar, 1.18; 95% CI, 0.59–2.38; HR for patients without LV scar, 1.00; 95% CI, 0.39–2.53, *p* for interaction = 0.79), despite a worse overall prognosis in the patients with LV scar. Thus, the increased risk associated with LV scar may not be associated with shockable ventricular arrhythmias. Furthermore, the fact that arrhythmic events occurred more often in patients with scar suggests that arrhythmic burden does not necessarily entail a net survival benefit from ICD in this population, hinting to alternative potential mechanisms as observed in ischemic cardiomyopathy (153).

### Prediction of Response to Cardiac Resynchronization Therapy

We have already discussed the role of echocardiography in identifying dyssynchrony and optimizing CRT therapy. Apart from echocardiography, CMR is also useful in the identification of patients who are likely to respond to CRT. In the context of ventricular dyssynchrony due to left bundle branch block, the LV free wall generally presents the longest delay activation time and thereby represents the target region for lead placement and electric stimulation (154). The gold-standard CMR technique for assessment of myocardial motion and deformation is CMR tagging (155). With this technique, the myocardium is tagged with markers, which are then traced throughout the cardiac cycle, providing information on myocardial displacement and strain. One of the disadvantages of this technique is that it involves complex postprocessing of the acquired information, and therefore it is not readily applicable to routine practice. Alternatively, short-axis cine imaging can be used to assess radial wall motion in order to quantify dyssynchrony (156). It is important to highlight that both TTE and CMR measures of dyssynchrony should not be used in isolation; they should rather serve as adjuncts to patient selection for CRT.

The clinical response to CRT depends on optimal lead position and viable cardiac muscle to be depolarized. It is reasonable to hypothesize that LGE (which represents myocardial fibrosis) can help predict clinical response to CRT and guide the lead placement away from areas of scar tissue. A recent CMR study suggested that a scar in the vicinity of RV lead may result in suboptimal LVRR (157). A scar close to the LV lead may be equally associated with poor LV resynchronization and prolonged QRS complex. Even more alarming were the results of one study which reported that LV lead positions over scar were associated with a higher risk of cardiovascular death (HR, 6.34; *P* < 0.0001) or hospitalizations for HF (HR, 5.57; *P* < 0.0001), compared with LV lead positions over viable myocardium (158). An intermediate risk of fulfilling these endpoints was observed when LV lead implantation was not guided by LGE location.

### Heart Transplant Recipients—Screening for Acute Rejection

Early detection of cardiac allograft rejection is of vital importance in post-transplant care. The current gold standard for diagnosis is endomyocardial biopsy (EMB), although this has the disadvantages of invasive risk, sampling error, and inter-operator variability.

CMR is attractive for rejection surveillance as it can achieve tissue characterization and detect myocardial inflammation (edema). T2 imaging has been most widely used. In a study which compared the diagnostic performance of CMR vs. EMB for acute rejection, CMR was found to have high sensitivity (93%) and high negative predictive value (98%), indicating that it may be possible to screen transplant recipients with CMR before performing EMB (159). Another study (160) reported that T2 relaxation time measured by T2 mapping is significantly higher in grade 2 rejection (the grade at which immunosuppressive therapy is generally augmented), compared with grade 0 or 1, and in grade 3 rejection compared with grade 2. A T2 relaxation time of ≥56 ms detected moderate acute rejections (≥grade 2) with a sensitivity of 89% and specificity of 70% (*p* < 0.0001). Notably, a T2 relaxation time of ≥56 ms in patients without ≥grade 2 rejection at baseline predicted the subsequent occurrence of ≥grade 2 rejection within the following 3 months, with sensitivity of 63% and specificity of 78% (*p* = 0.001). Combining myocardial T2 relaxation time alongside ECV may further improve the diagnostic accuracy of CMR in diagnosis and differentiating the diverse stages of acute cardiac allograft rejection (161, 162).

## Future Developments

Diffusion tensor CMR (DT-CMR) is a novel technique for in-depth phenotyping through non-invasive interrogation of the three-dimensional heart microarchitecture (163, 164). DT-CMR evaluates myocardial microstructure using helix angle (HA) and absolute angulation of the second eigenvector (E2A) to assess cardiomyocyte and sheetlet orientation, respectively. In healthy subjects, the sheetlets align more wall-parallel in diastole and more wall-perpendicular in systole. However, in DCM patients, the sheetlets have altered systolic confirmation and reduced mobility (E2A is reduced in systole, indicating that the sheetlets are stuck in a more diastolic orientation and fail to reorient as expected) (165). A recent study focused on recovered DCM subjects with symptomatic and structural improvement (entirely recovered LV size and normal LVEF). Systolic E2A and sheetlet mobility were found to remain significantly reduced in all recovered DCM subjects compared with the controls, suggesting persistent abnormalities at the myocardial microstructure level despite normalization of clinical and imaging parameters as well as symptom resolution (166). This highlights a potential role of DT-CMR in the differentiation between recovery and remission of DCM and the identification of patients at risk of relapse, which could have significant implications on long-term treatment and follow-up of patients with recovered LV function.

Finally, four-dimensional flow CMR is an emerging technology used to visualize and quantify intra-cardiac blood flow. Various studies have used it to compare the LV hemodynamic forces of normal individuals with that of DCM patients. Eriksson et al. reported that the LV hemodynamic filling forces and the diastolic flow routes through the LV of DCM patients are heterogeneous in direction and magnitude (167, 168). It was noted that these changes in the flow route and energetics are seen in clinically compensated mild LV dysfunction, and it was therefore hypothesized that they may be useful as subclinical markers of LV dysfunction.

## Conclusion

Overall, correct diagnosis, investigation of causes, and risk stratification of DCM patients remain a challenge. A wide variety of constantly evolving imaging tools is at the disposal of clinicians, and they can help tailor treatment to the needs of this patient population. Among imaging modalities, CMR constitutes a versatile technique that can visualize numerous aspects of structural and functional information of the failing heart. As future guidelines are expected to upgrade CMR into an integral examination for DCM patients, intensive research on novel imaging sequences and the outcomes of CMR-guided treatment decisions is warranted with the overarching aim to expand the applications of this technique and to transform the care of this patient population in the near future.

## Author Contributions

PM and GG wrote this review paper, with support from SF, DK, and FN. PGM supervised the writing of the manuscript and provided valuable feedback and guidance. All the authors contributed to manuscript revision and read, and approved the submitted version.

## Conflict of Interest

The authors declare that the research was conducted in the absence of any commercial or financial relationships that could be construed as a potential conflict of interest.
